# Application of advanced very high-resolution radiometer (AVHRR)-based vegetation health indices for modelling and predicting malaria in Northern Benin, West Africa

**DOI:** 10.1186/s12936-024-04879-1

**Published:** 2024-03-15

**Authors:** Gouvidé Jean Gbaguidi, Mouhamed Idrissou, Nikita Topanou, Walter Leal Filho, Guillaume K. Ketoh

**Affiliations:** 1 West African Science Service Centre on Climate Change and Adapted Land Use (WASCAL), Faculty of Human and Social Sciences, Department of Geography, University of Lomé, Lomé, Togo; 2 Kaba Laboratory of Chemical Research and Application (LaKReCA), Department of Chemistry, Faculty of Science and Technic of Natitingou, University of Abomey, Abomey, Benin; 3 Research and Transfer Centre Sustainability and Climate Change Management, Faculty of Life Sciences, Hamburg University of Applied Sciences, Ulmenliet 20, 21033 Hamburg, Germany; 4 École Polytechnique de Lomé, University of Lomé, Lomé, Togo; 5 Laboratory of Ecology and Ecotoxicology, Department of Zoology, Faculty of Sciences, University of Lomé, 1BP: 1515 Lomé, Togo

**Keywords:** AVHRR, Vegetation health, Malaria, Forecasting, Benin

## Abstract

**Background:**

Vegetation health (VH) is a powerful characteristic for forecasting malaria incidence in regions where the disease is prevalent. This study aims to determine how vegetation health affects the prevalence of malaria and create seasonal weather forecasts using NOAA/AVHRR environmental satellite data that can be substituted for malaria epidemic forecasts.

**Methods:**

Weekly advanced very high-resolution radiometer (AVHRR) data were retrieved from the NOAA satellite website from 2009 to 2021. The monthly number of malaria cases was collected from the Ministry of Health of Benin from 2009 to 2021 and matched with AVHRR data. Pearson correlation was calculated to investigate the impact of vegetation health on malaria transmission. Ordinary least squares (OLS), support vector machine (SVM) and principal component regression (PCR) were applied to forecast the monthly number of cases of malaria in Northern Benin. A random sample of proposed models was used to assess accuracy and bias.

**Results:**

Estimates place the annual percentage rise in malaria cases at 9.07% over 2009–2021 period. Moisture (VCI) for weeks 19–21 predicts 75% of the number of malaria cases in the month of the start of high mosquito activities. Soil temperature (TCI) and vegetation health index (VHI) predicted one month earlier than the start of mosquito activities through transmission, 78% of monthly malaria incidence.

**Conclusions:**

SVM model D is more effective than OLS model A in the prediction of malaria incidence in Northern Benin. These models are a very useful tool for stakeholders looking to lessen the impact of malaria in Benin.

## Background

Malaria is one of the most dangerous infectious illness in sub-Saharan Africa (SSA). The transmission of malaria is influenced by socioeconomic and environmental factors. The environment plays a defining role in the health outcomes of any society. Unfortunately, the environment has a particularly detrimental impact on health for the majority of developing countries [1]. Infection with malaria is influenced by temperature, precipitation, and altitude [2, 3]. The burden of mortality and morbidity is worse in poor countries and among the most disadvantaged in these countries [1]. The transmission of malaria is complex and involves a range of hydro climatological, biological, and environmental processes [4]. The high degree of nonlinearity in these processes makes it challenging to anticipate and combat malaria [5].

Despite national and international efforts, malaria remains a significant public health problem in many countries, including Benin [6]. The prevalence of malaria in the WHO African Region in 2020 is estimated to account for 95% of cases and 96% of deaths worldwide from malaria. The number of malaria cases has increased since 2021, reaching 234 million [7]. The lack of accurate information on the actual burden of malaria, the risk of *Plasmodium* transmission, and its geographical distribution hinders the effectiveness of existing healthcare systems [8].

In Benin, as in several other sub-Saharan African nations, malaria stands as the primary contributor to both mortality and morbidity. The reported annual malaria cases in 2021 amounted to 3,163,648, indicating a marginal increase of 1.4% from the previous year. This disease accounted for 44% of medical consultations and 31% of hospitalizations [9].

Meteorological and rainfall gauge stations are limited in Benin, making obtaining historical and spatially continuous observations of climatic/environmental variables difficult. Developing reliable modelling frameworks to integrate climate and infectious disease predictions is a major challenge in West Africa, particularly in Benin, which has limited rainfall and meteorological stations [10]. Remote sensing satellites can be an alternative to obtaining proxy data from satellite websites. Remote sensing data-based vegetation health indices help experts understand environmental factors and malaria transmission [11]. Vegetation conditions play a role in stimulating mosquito activities, thus indirectly influencing the transmission of malaria. Incorporating the vegetation conditions into this study will yield valuable insights into the malaria risk, representing a crucial step in the malaria control process.

Through advanced seasonal weather forecasting that draws upon established associations between weather/climate and infection/transmission conditions, conditions conducive to disease outbreaks can be identified months in advance, providing time to implement effective population health responses. Early malaria detection outbreaks remain an important tool to prevent diseases in SSA [12].

To track malaria epidemics in Northern Benin, we attempted to use vegetation health indices (VH) created from the Advanced Very High-Resolution Radiometer (AVHRR) launched on the NOAA series of operational polar-orbiting satellites. To be more accurate, an effort was made to develop malaria incidence-VH models using principal component regression (PCR), ordinary least squares (OLS) and support vector machine. OLS is a linear regression method that provides coefficients for predictor variables, but can be sensitive to outliers and multicollinearity. SVM captures nonlinear relationships using kernel functions, minimizing outliers and preventing overfitting. PCR reduces multicollinearity. These models were used for malaria epidemic forecasts [13].

## Methods

### Presentation of the study area

**Fig. 1:**
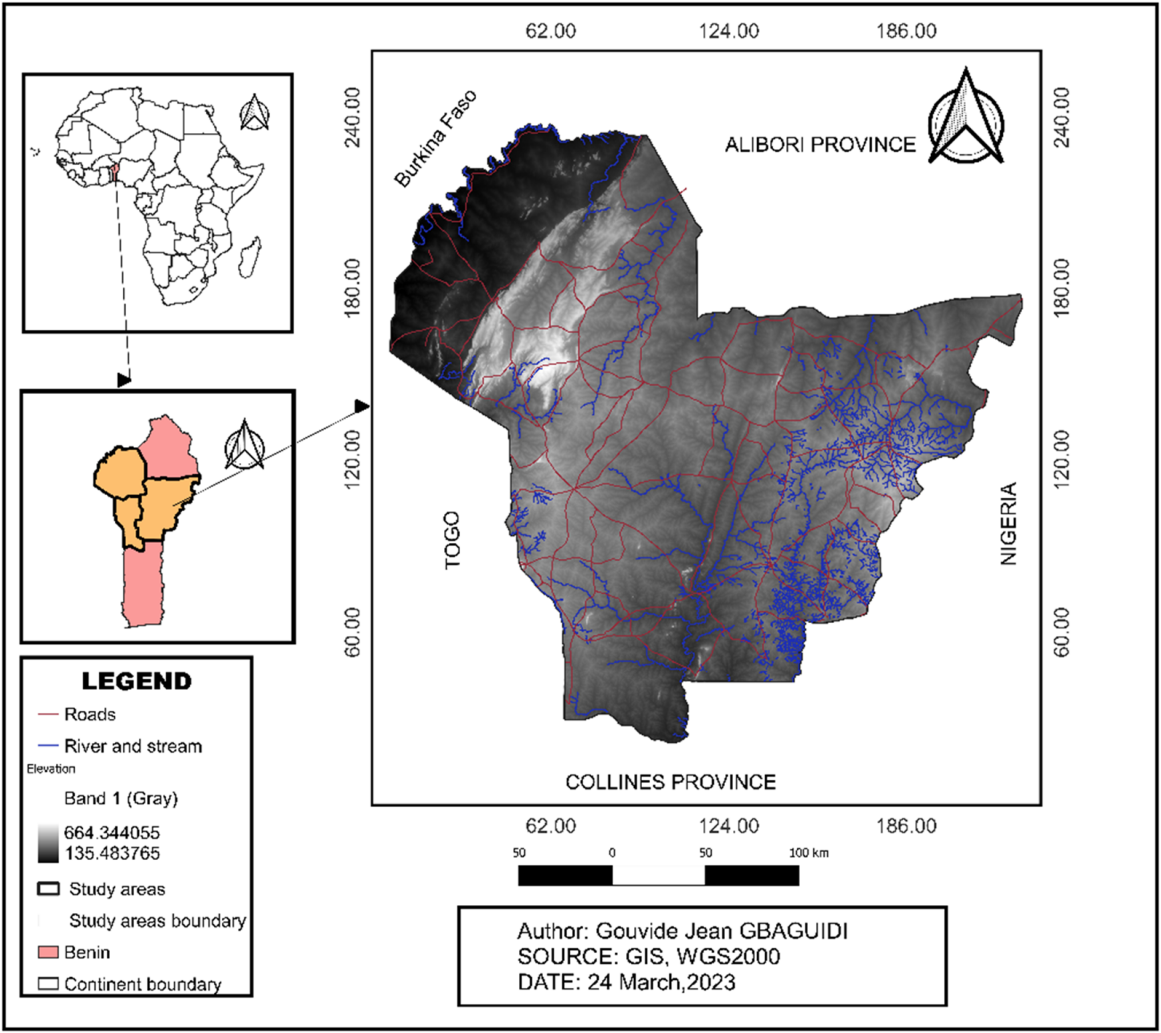
Map of the study area

**Fig. 2:**
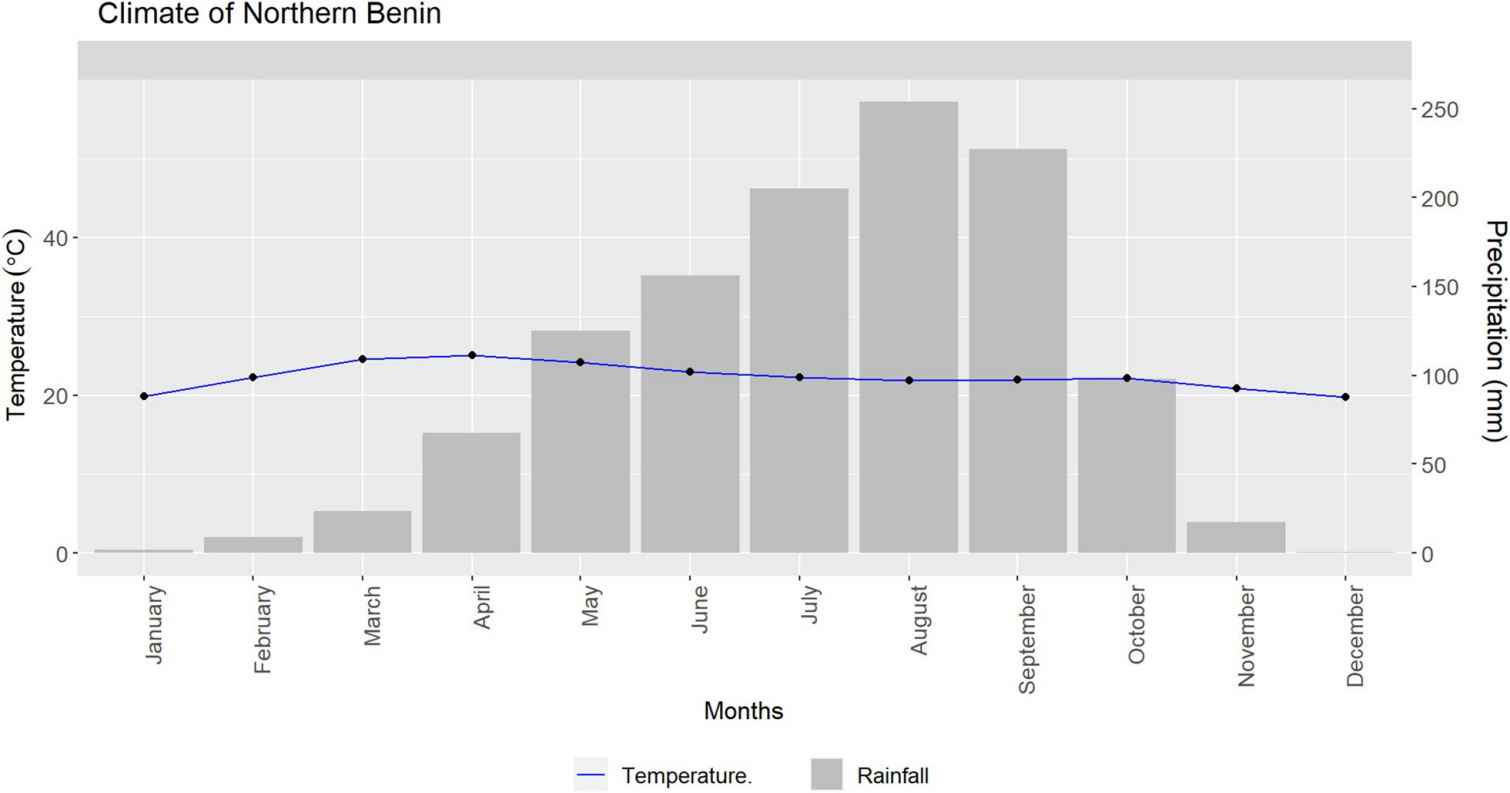
Climate of Northern Benin, West Africa

Atacora, Donga, and Borgou are the provinces of the study area situated in Benin's northern region (Fig. 1). Northern Benin receives between 200 and 300 mm of rainfall per month during the rainy season (July September) [14]. Northern Benin has a dry season and a wet season. The rainy season lasts from May–October. Rainfall is maximal (253.61 mm) in August and minimal (1.90 mm) in January. The dry season is from November–April when the ‘Harmattan’ winds blow in from the northeast, bringing air from the Sahara Desert (Fig. 2). The population of the study area in 2021 is 3,606,063 (RGPH4). The 2016–2017 period was marked by a slight decrease in the number of malaria cases. This highlights the effort made by the government and its partners to reduce the burden of malaria.

### Data collection

#### Endogenous variables

Monthly malaria cases in each district were collected from 2009 to 2021 at the Ministry of Health of Benin. These data include the entire population confirmed to be infected by malaria. When malaria is identified in a patient using rapid diagnostic testing or microscopy in a laboratory, the diagnosis is made [15].

#### Exogenous variables

To assess the impacts of land cover change, vegetation health conditions from remote sensing data were used. Numerous studies have leveraged satellite data to investigate infectious diseases, including malaria [16]. There are several vegetation indices available, with the normalized difference vegetation index (NDVI) being the most often used index in some human health studies. However, relying solely on the NDVI may not encompass all the relevant environmental parameters associated with malaria. Research conducted by Kogan and colleagues has demonstrated that the vegetation health indices is better suited for detecting malaria risk [17].

The AVHRR onboard the National Oceanic and Atmospheric Administration (NOAA) series of satellites was used to calculate the weekly brightness temperature (BT) and the Normalized Difference and Vegetative Index (NDVI) from 2009 to 2021 [17]. BT was converted to the Vegetation Condition Index (VCI), the Temperature Condition Index (TCI) and the Vegetation Health Index (VHI), which are useful indices for the estimation of vegetation health and malaria monitoring. Weekly TCI, VHI and VHI were connected to the equivalent month’s malaria case data.

### Data processing

#### Public health data

Malaria transmission and consequences are influenced by climate and environmental factors [18]. The time series data are divided into two parts: deterministic for the first group (Ysp), which represents socioeconomic/policy issues, and random for the second group (Yw). The percentage of malaria cases in Yw is determined by changes in the weather. The observed malaria cases in northern Benin can be expressed by (1).

A linear trend was derived using the time series of the percentage of malaria cases instances of malaria to show the interannual variation in malaria prevalence, which is probably partly brought on by interannual changes in weather patterns. As a ratio of the actual malaria cases to the estimated trend, the weather-related variability around the trend was expressed (2) [19, 20]. Due to yearly and seasonal weather variations, Yw represents the divergence (anomaly) of malaria episodes from the trend. Yw is an effective comparative indicator of malaria cases.

1 $$DY=Yw+Ysp$$

2 $$Yw=(Ysp*100)/Yt$$

where $Yt$ represents the estimated trend of the percentage of malaria cases over time.

#### Satellite data: vegetation health indices

Using a functioning satellite, the goal is to identify early environmental conditions that favour mosquito growth and the spread of malaria. The principle to construct VH indices stems from the property of green vegetation to reflect/emit solar radiation in the VIS, NIR and IR wavelengths. If vegetation is healthy, it reflects little radiation in the VIS (due to high chlorophyll absorption of solar radiation) and much in the NIR (due to scattering of light by leaf internal tissues and the low absorption by pigments, water and other leaf constituents), resulting in a high NDVI. The global vegetation index (GVI) was developed from the reflectance/emission observed by the Advanced Very High-Resolution Radiometers (AVHRR) of the NOAA polar‐orbiting satellite in the visible (VIS), near-infrared (NIR) and infrared (IR) wavelengths [17]. In developing the GVI, the measurements were spatially sampled from 16 km^2^ (National Area Coverage–NAC) to 16 km^2^ and from daily observations to a seven‐day composite [21]. The VIS and NIR reflectance was pre- and post‐launch calibrated, and NDVI was calculated by the relation.

3 $$NDVI=\frac{NIR-VIS}{NIR-VIS}$$

The IR measurements at wavelengths of 10–11 μm is converted to BT. The high-frequency noise of the NDVI and BT related to variable transparency of the atmosphere, bidirectional reflectance, orbital drift, randomness, and others was already removed from the data by statistical techniques [22]. The 2009–20021 weekly NDVI and BT data were collected for each 16 km^2^ pixel of the study area (Northern Benin), which is the most infected by malaria. Finally, the weekly minimal and maximal values of NDVI and BT of the investigated period were computed.

From the values of the NDVI and BT, we calculated three indices (Eqs. 4, 5 and 6): the vegetation condition index (VCI) characterizing moisture, the thermal condition index (TCI) characterizing the soil temperature and the vegetation health index (VHI) [16].

4 $$VCI=100*\frac{NDVI-NDVImin}{NDVImax-NDVImin}$$

5 $$TCI=100*\frac{BTmax-BT}{BTmax-BTmin}$$

6 $$VHI=a*VCI+(1-a)*TCI$$

where NDVI, NDVImax, and NDVImin (BT, BTmax, and BTmin) are the no noise monthly NDVI and BT, their multi‐year absolute maximum and minimum, respectively. *a* is a coefficient quantifying the share of VCI versus TCI contribution in overall vegetation health. Because this share is not known for a specific location, it is assumed that the shares are equal, *a* = 0.5 [23]. All three indices are scaled to range from 0 to 100 corresponding to variation in weather conditions from extremely stressed to the most favourable vegetation, respectively. To examine the relationship between vegetation health and malaria incidence, Pearson correlation and regression analysis were used in this study. The dynamics of vegetative health during the lowest and greatest malaria incidences motivated us to examine the relationship between malaria incidence and vegetation health indices (TCI, VCI, and VHI). As malaria incidence is available on a monthly scale, monthly malaria incidence was connected with weekly vegetation health index data.

### Machine learning

To investigate whether VH is highly correlated with malaria incidence in favourable weather conditions for the multiplication and intensification of mosquito activities, models were built using the premise that they would only contain variables that have a correlation coefficient of Yw with TCI, VCI, and VHI greater than 0.55 (p < 0.05). On the basis of these ideas, the following set of independent variables, four models were chosen with the following set of independent variables: (A) mean VCI for weeks 19–21 (VCI19-21), (B) mean VHI for weeks 49–52 (VHI49-52), (C) the same variable in A and mean VHI for weeks 29–32 (VHI29-32), (D) the same variable in (B) and TCI for weeks 11–13 (TCI11-13).

Support vector machine (SVM), ordinary least squares (OLS), and principal component regression (PCR) were applied to regress the incidence of malaria (Yw) regulated by weather conditions. Tables 1, 2 and 3 present a summary of the various built models and the different metrics used to assess the performance and validation of each model.

**Table 1:** Ordinary least squared regression (OLS)

Models	Shapiro‒Wilk	RMSE	MSE	MAE	R-squared predicted
(A) = 4.85 + 0.28*VCI19-21	0.77	2.43	5.89	1.53	0.75
(B) = −8.23 + 0.44*VHI49-52	0.88	4.70	22.05	3.82	0.35
(C) = 33.66 + 0.09VCI19-21–0.35*VHI29-32	0.17/0.02	7.47	55.82	5.70	0.29
(D) = 6.67–0.05*TCI11-13 + 0.21*VHI49-52	3.17E–05	3.46	11.98	2.79	0.27

**Table 2:** Support vector machine regression (SVM)

Models	RMSE	MSE	MAE	R-squared predicted
(A)Yw = f(VCI19-21)	3.04	9.25	1.88	0.60
(B)Yw = f(VHI49-52)	4.47	20.00	3.33	0.41
(C) = Yw = f(VCI19-21,VHI29-32)	6.93	48.02	4.50	0.41
(D)Yw = f(VHI49-52,TCI11-13)	1.91	3.65	1.61	0.78

**Table 3:** Principal component regression (PCR)

Models	RMSE	MSE	MAE	R-squared predicted
(C) PCR(VCI19-21,VHI29-32)	6.23	38.79	4.29	0.53
(D) PCR (VHI49-52, TCI11-13)	3.46	11.98	2.79	0.27

To define a training set and test set, as well as to train the model and assess how well it predicts, A random sampling method was used to define a training set and test set to train the model and to evaluate the performance of models to predict. 90% of the data for training and 10% for testing a predictive model were randomly selected. Support vector machine (SVM), ordinary least squares (OLS) and principal regression (PCR) algorithms represented by Eqs. 7, 8 and 9, respectively were applied to construct the malaria outbreak warning model. SVM and OLS were used to examine the influence of single vegetation health indices on the incidence of malaria. To investigate the impact of multiple vegetation health indices on the occurrence of malaria, SVM, OLS and PCR were employed.

7 $$Malari{a}_{incidence}= \mathrm{\alpha }+\upbeta 1 *\mathrm{ VCI }+\upbeta 2 *\mathrm{ TCI }+\upbeta 3 *\mathrm{ VHI}$$

with α representing the intercept, and β1, β2, and β3 are the coefficients associated with the independent variables VCI, TCI, and VHI, respectively.

8 $$Malari{a}_{incidence}= \alpha + \beta 1 * VCI + \beta 2 * TCI + \beta 3 * VHI$$

where α is the intercept, and β1, β2, and β3 are the coefficients associated with the independent variables VCI, TCI, and VHI, respectively.

9 $${{\text{Malaria}}}_{{\text{incidence}}}=\mathrm{ \alpha }+\upbeta 1 *\mathrm{ PC}1 +\upbeta 2 *\mathrm{ PC}2 +\upbeta 3 *\mathrm{ PC}3$$

where α is the intercept and β1, β2, and β3 are the coefficients corresponding to PC1, PC2, and PC3, respectively, of the principal components.

To identify the optimum forecasting algorithm [24], the mean absolute error (MAE), mean square error (MSE), root mean square error (RMSE), and R-squared value on a validation dataset through random sampling of the proposed models (SVM, OLS, and PCR) were computed for the prediction of malaria incidence. The accuracy of the best-selected models was assessed based on the bias and the standard deviation of the observed malaria incidence and the predicted incidence. The R software version 4.3.2 was used to extract and compute the values of VHI, VCI and TCI [25].

## Results

### Dynamics of malaria

**Fig. 3:**
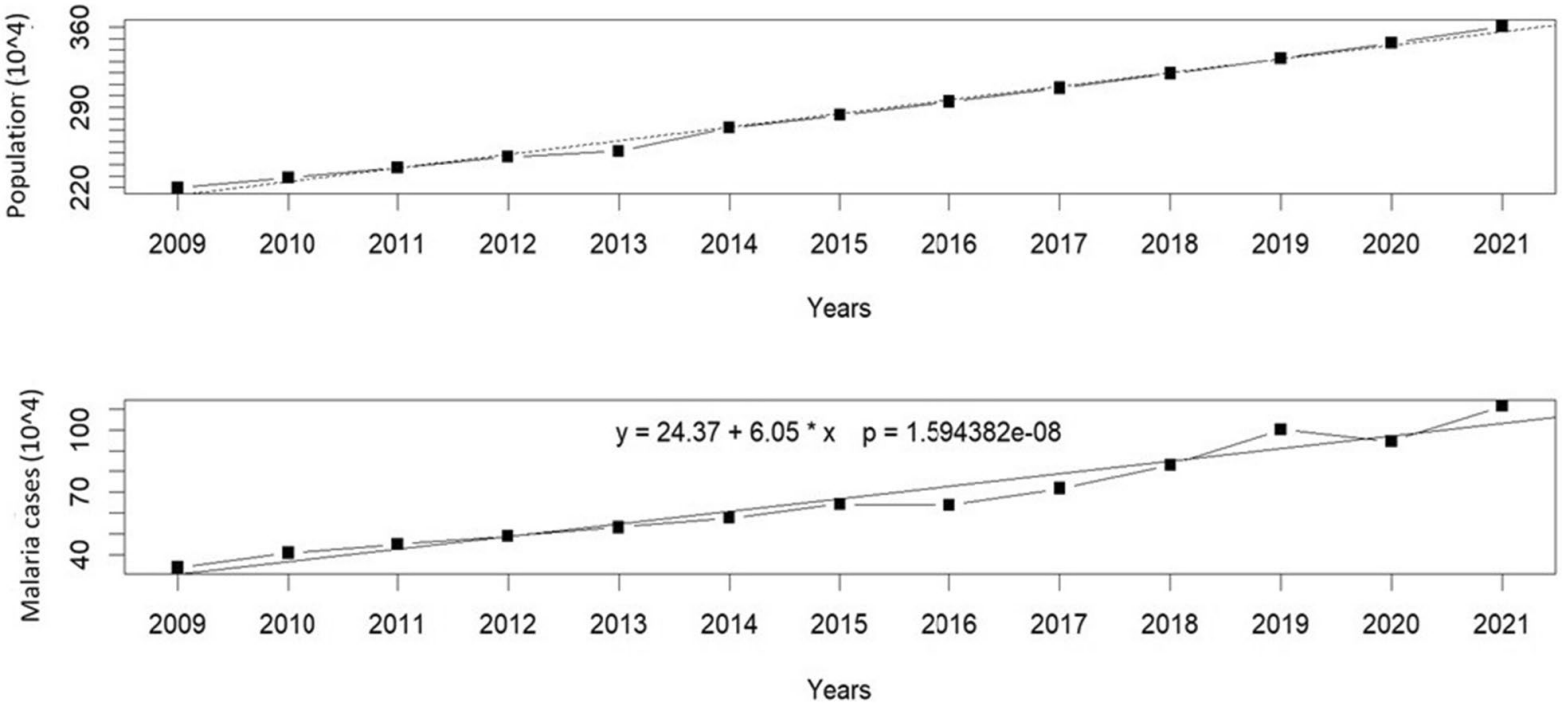
Time series for the population and the malaria cases

Figure 3 displays the annual time series number of malaria cases in Northern Benin from 2009 to 2021. The analysis examines the relationship between the number of Malaria cases and the passage of time, represented by the variable 'Year'. The regression model revealed a statistically significant positive relationship between the two variables (Ysp = 24.37 + 6.05 * Year, p < 0.001). This indicates that when considering a mean of malaria cases of 66.74, the yearly increase in malaria cases in percentage is estimated to be approximately 9.07% based on the regression analysis. There was a significant association between year and malaria cases (p = 1.59e−08). From 2017 to 2021, the number of reported cases has increased, except in 2020 (Fig. 3). Since there has been an increase in the population, the number of cases of malaria has also increased (p = 4.034e-09, *R*^*2*^ = 0.98). In 2009, the weather-related variability of malaria cases (Yw) was 33.82% and 111.40% in 2021, respectively, the lowest malaria case year and the most severe malaria outbreak (Fig. 3). This finding suggests a significant annual rise in malaria incidence, underscoring the need for effective control and prevention measures to address the escalating public health challenge posed by malaria.

### Vegetation health dynamics in the lowest and extreme malaria years

**Fig. 4:**
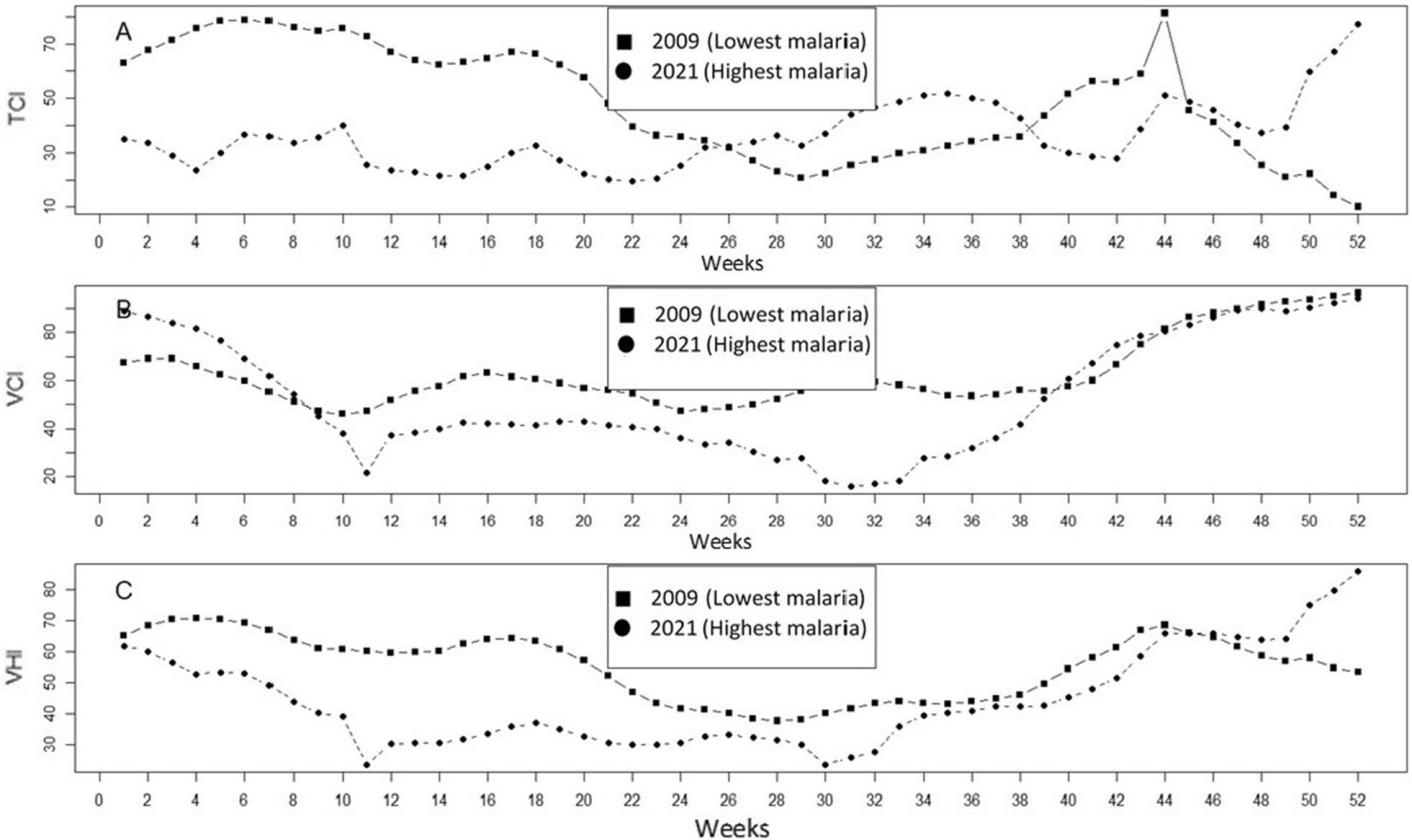
Dynamics of vegetation (VCI), temperature (TCI) condition and vegetation health (VHI) index for the two years of number of malaria cases: Lowest (2009) and extreme (2021) in the 2009–2021 period

To have a basic grasp of whether weather conditions indicated using VH indices provide any helpful indicators about the likelihood that malaria will develop, the year with the lowest (2009) and the highest (2021) number of malaria cases was chosen. Figure 4 presents the dynamics of vegetation (VCI), temperature (TCI) condition and vegetation health (VHI) index for the two years of the extreme number of malaria cases: lowest (2009) and extreme (2021) in the 2009–2021 period. The dynamics of VH indices in these years were compared in the selected malaria-prone areas. These conditions, as expressed through VH indices, have some useful signals about the potential for the development of malaria. The number of malaria cases is high from May to November (rainy season) and low in the dry season (December to April) for both years of malaria cases. The TCI is high (warmer weather) during the period (weeks 1–22 and week 38–47) and low (cooler condition) from (weeks 23–37 and weeks 48–52) with the worst malaria cases year (2009) and the opposite during the same period with the highest malaria cases (2021) in Fig. 4A. Mosquito activity is slowed down by warmer weather and stimulated by cooler weather.

The VCI and VHI both stimulate mosquito activity and malaria transmission throughout the rainy season (weeks 23–47), during the year with fewer cases and the year with more cases. In the dry season (weeks 48–22), the situation is the exact opposite in Fig. 4B and C.

### Correlation analysis

**Fig. 5:**
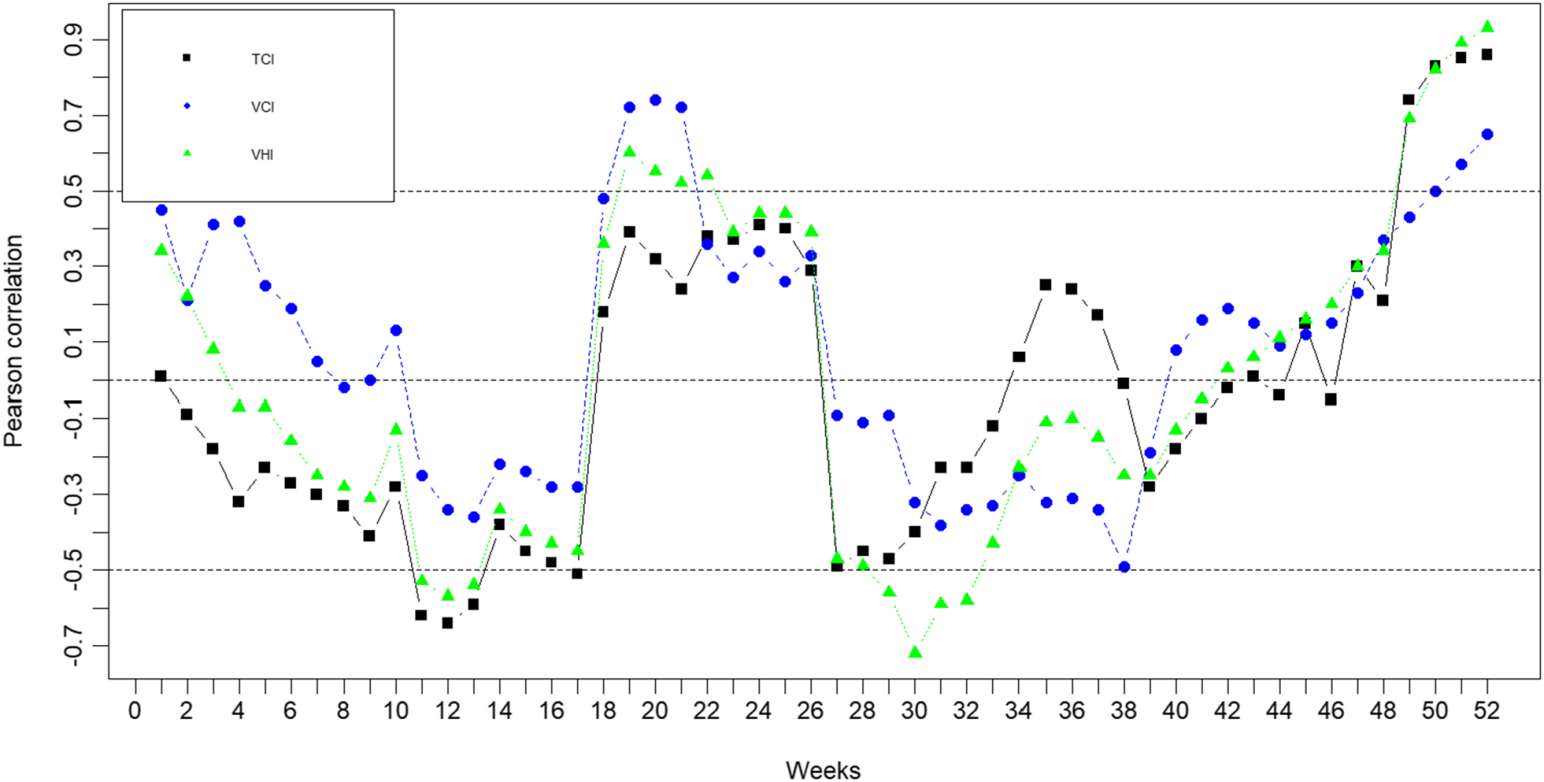
Correlation coefficient between the monthly malaria incidence index (Yw) with the weekly Temperature Condition Index (TCI), Vegetation Condition Index (VCI), and Vegetation Health Index (VHI)

Figure 5 displays the dynamics of the Pearson correlation between weekly vegetation health indices and monthly malaria incidence (Yw) over the period 2009–2021. The analysis of the figure reveals that the correlation between the TCI and malaria incidence ranges from − 0.64 to 0.86. The correlation is low (below 0.55) by the beginning of the dry season and rainy season during the period from January to half-March (weeks 1–10) and May to the end of November (weeks 14–48), respectively. The correlation between the incidence of malaria and the TCI increases from − 0.62 to − 0.59 during weeks 11–13, corresponding to the second mi-March to 30 April period. There is an inverse relationship between TCI and malaria incidence during this period. During December (weeks 49–52), corresponding to the start of the dry season, the correlation increases from 0.74 to 0.86, indicating a robust positive connection between TCI and malaria incidence starting at week 49. A high correlation was observed from the beginning and end of the dry season, indicating that hot weather conditions reduce mosquito activities through malaria infection. A TCI greater than 42 is harmful for the development of mosquitoes and indirectly their activities to infect humans.

The correlation between moisture (VCI) and the incidence of malaria started to increase from 0.72 to 0.74 in May (weeks 19–21), corresponding to the beginning of mosquito breeding sites and their activities. Weeks 51–52, corresponding to the last two weeks of December, were associated with elevated mosquito activities. Moisture stimulates the development and activities of mosquitoes to transmit malaria. The high correlation observed in the May and December rhythms with the results of Fig. 4. A VCI above 55 restrains the activities of mosquitoes.

Concerning the relationship between the incidence of malaria and VHI, the correlation starts to increase from weeks 19–52, corresponding to the period of May to December. This period is associated with intense activity of mosquitoes associated with elevated malaria incidence. It is important to emphasize that the correlation in weeks 29–32 (June) is negative. This could be due to a number of factors, such as increased vegetation cover providing more shade and reducing mosquito breeding habitats or healthier vegetation leading to higher biodiversity that includes natural predators of mosquitoes. The VHI exhibits a pattern resembling that of the TCI and VCI, exhibiting a variable association in the early weeks and a significant positive correlation in the latter weeks.

Based on the correlations found, it appears that more favourable weather, as indicated by higher TCI, VCI, and VHI values, may help boost malaria transmission. This discovery emphasizes the significance of considering weather patterns and vegetation health when analysing and controlling malaria epidemics. It also implies the possibility of using the monitoring of vegetation health indices as an early warning system for anticipating and reducing malaria transmission in impacted areas.

### Machine learning

The analysis of Table 1 presenting OLS regression models reveals that Model A seems to have the best performance and the lowest prediction error based on the criteria given. The predicted values are fairly close to the observed values, as indicated by the fact that it has the lowest root mean square error (RMSE), 2.43. The model also has a high R-squared value of 0.75, which shows that the predicted and actual values fit together well. In addition, the Shapiro‒Wilk test (p = 0.77) confirmed that the residuals were normally distributed. The studentized Breusch‒Pagan test yielded a P value of 0.27, indicating that the variance of the errors in the linear regression model is consistent across all levels of the independent variables, which is a desired characteristic of a well-fitted linear regression model.

Models B, C, and D, on the other hand, show greater prediction errors with RMSE values of 4.70, 7.47, and 3.46, respectively. The residuals are not normally distributed, as shown by Model D's lowest Shapiro‒Wilk value (3.17E-05). The largest average discrepancy between the anticipated and actual values is shown by Model C, which also has the greatest MSE and MAE values.

Concerning the Table 2 displaying SVM regression models, model D appears to be the model with the lowest error for estimating the prevalence of malaria. Since it has the lowest RMSE (1.91), the predicted and observed values are the closest in this case. The model's high R-squared score of 0.78 further demonstrates the model's ability to accurately predict both actual and forecasted values. The prediction errors are higher for Models B and C, with RMSE values of 4.47 and 6.93, respectively. Model A has a high R-squared value of 0.60 with high prediction errors (3.04). Therefore, Model D predicts the incidence of malaria well and fits the data better than Model A.

Based on the provided criteria in Table 3 showing the PCR regression model, Model B seems to be the model in the collection that does the best job of accurately forecasting the prevalence of malaria. The model has an RMSE of 3.46 and an R-squared value of 0.27, and the variables VHI49-52 and TCI11-13 are included.

Overall, the support vector machine (model D) and Model A from ordinary least squares regression are the best models to predict the monthly incidence of malaria with the least amount of error. Model D built with PCR has a very low R-squared value and will not add any further details to the malaria prediction.

**Fig. 6:**
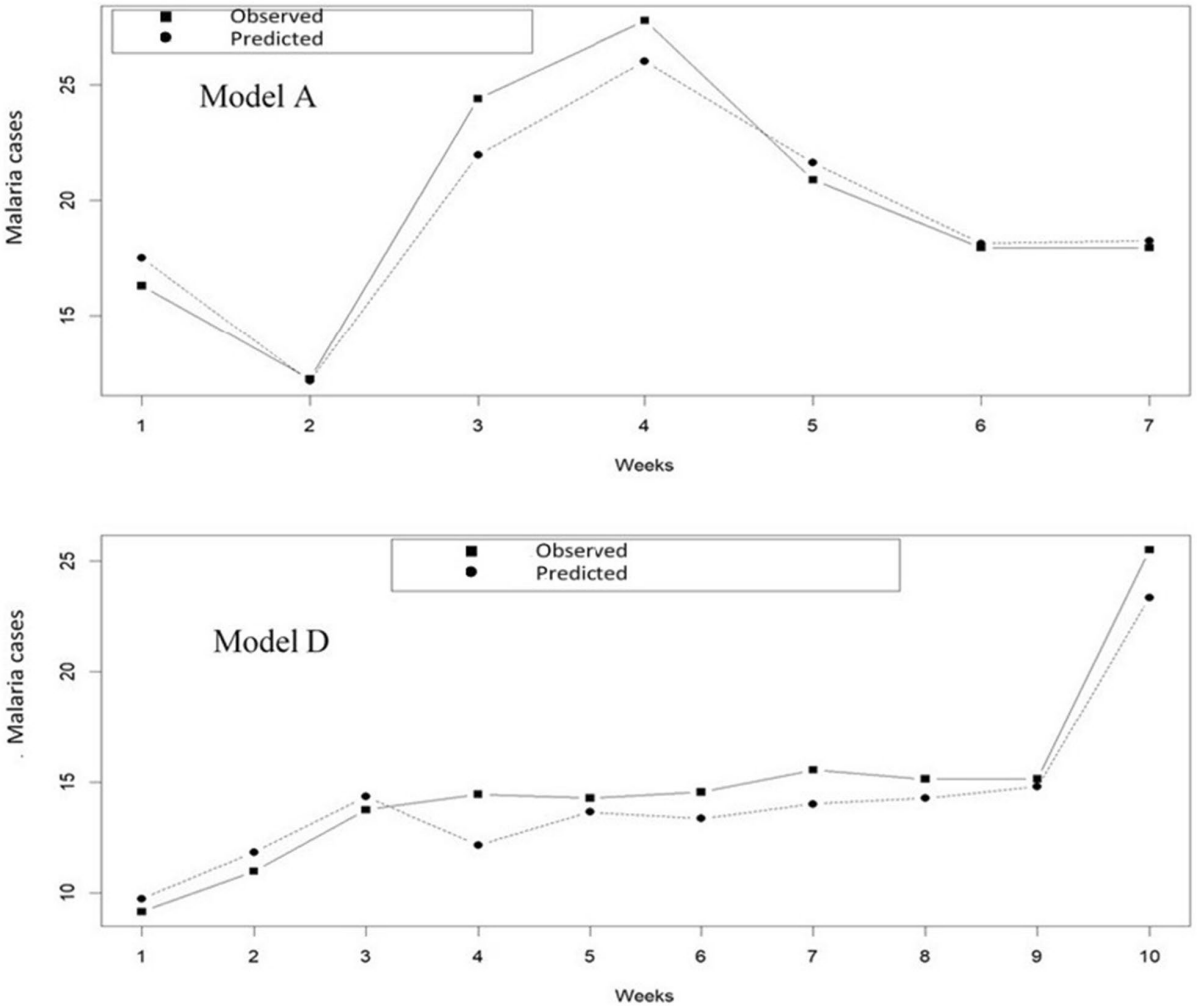
Observed and simulated percent malaria cases from Model A and Model D of the random sampled test data

By analysing Table 4 and Fig. 6, the performance of models A and D in predicting malaria incidence using different metrics was compared. The mean value of the observed malaria incidence for model A is 19.64, while the mean value of the predicted malaria incidence is 18.81, resulting in a bias of 0.83. Concerning model D, the mean value of the observed malaria incidence is 14.85, while the mean value of the predicted malaria incidence is 14.65, resulting in a bias of 0.196. This means that the predicted values of model D are closer to the observed values, indicating that model D may be more accurate in predicting malaria incidence (Fig. 6).

**Table 4:** Observed and independently simulated percentages for models A and D from OLS regression and SVM regression, respectively

Models	Metrics	Observed	Simulated	Bias
A	Mean	19.64	18.81	0.83
	Standard_Deviation	5.20	3.55	2.46
D	Mean	14.84	14.65	0.20
	Standard_Deviation	4.27	3.61	2.00

Moreover, the standard deviation of the observed and predicted malaria incidence is also an important metric to consider. For model A, the standard deviation of the observed malaria incidence is 5.20, while the standard deviation of the predicted malaria incidence is 3.55, resulting in a difference of 2.46. For model D, the standard deviation of the observed malaria incidence is 4.265, while the standard deviation of the predicted malaria incidence is 3.61, resulting in a difference of 2.00. This indicates that model D is better at predicting values that are closer to the true values, as it has a lower difference between the standard deviation of the observed and predicted malaria incidence on the one hand and a lower bias.

## Discussion

Vegetation health conditions have a significant impact on mosquito reproduction and survival, which are the main carriers of the disease. The state of vegetation is frequently tightly correlated with environmental factors, including temperature, humidity, and rainfall, which might affect the number of mosquito breeding sites.

In the northern part of Benin, the correlation between vegetation health and malaria incidence varies during the season. A significant correlation between the soil temperature and the incidence of malaria from the second half of March to April 15th and the last two weeks of December was found. From January to March, the soil temperature is high due to the high intensity of the air temperature during these months. A high value of TCI decreases mosquito activities through the transmission of malaria. A mean TCI value greater than 42 is not favourable for the reproduction or multiplication of mosquitoes. This is the main cause of the dry season's low malaria incidence. On the other hand, a TCI value ranging between 0 and 30 is a good preference for mosquitoes. In the wet season, the TCI at this scale and the low correlation obtained in the period of May to November (rainy season) could be explained by these TCI values [20]. found a significant correlation between the incidence of malaria and the TCI in Tripura, India. The author agreed that a low value of TCI reduces the number of infected people. The high correlation between soil moisture (VCI) and malaria incidence indicates high confidence that vegetation stimulates multiplication and increases the activities of mosquitoes in the rainy season. The high intensity of malaria observed in December is stimulated by VCI, which characterizes moisture. Throughout the rainy season, malaria incidence is strongly correlated with VHI, which measures the health and development of vegetation. This finding is consistent with the conclusion of the study of Kogan [21], who found that VH influences the incidence of malaria.

**Fig. 7:**
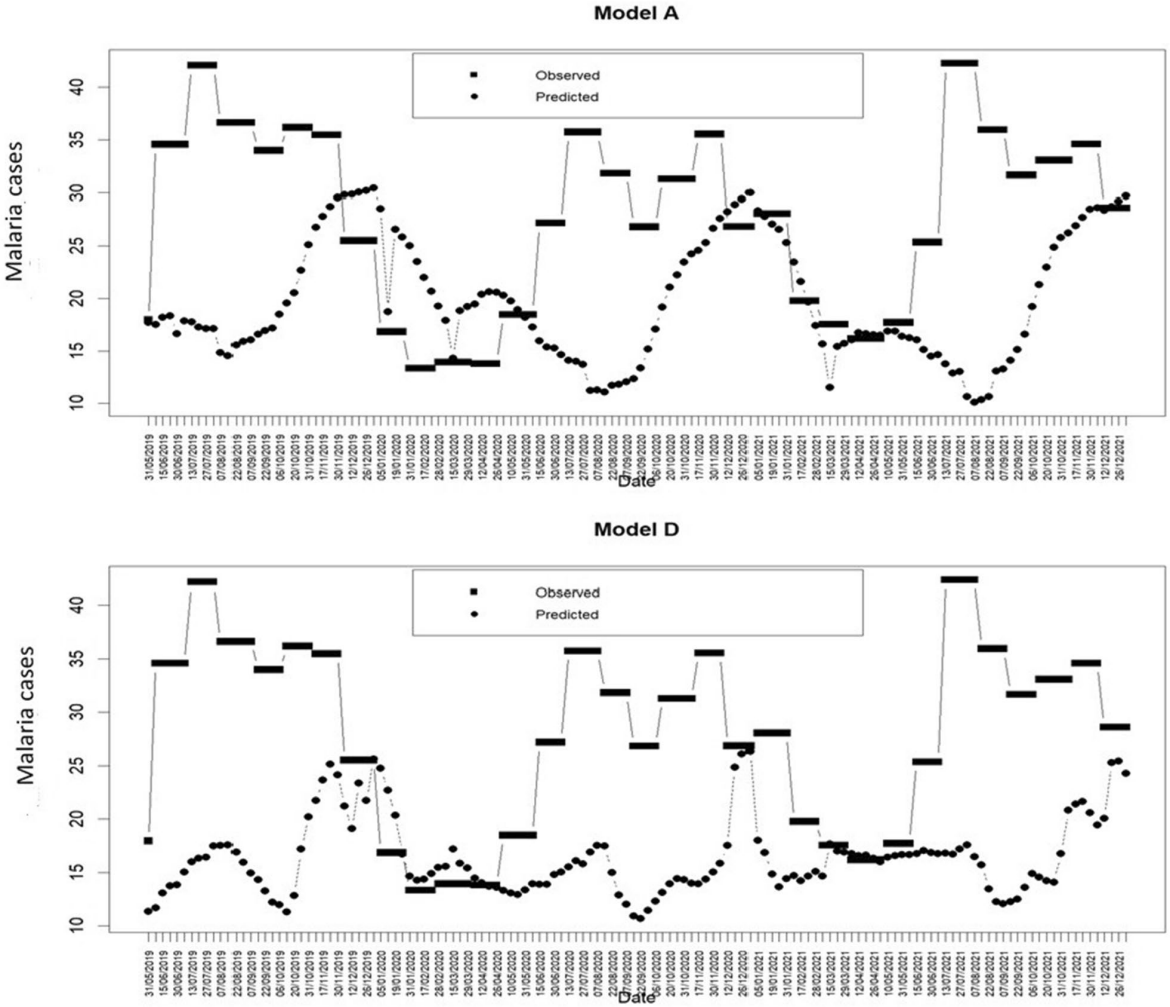
Time series of observed and simulated percent malaria cases from Model A and Model D for model validation with the historical sampled test data

Model D outperforms Model A in predicting malaria incidence. Model D predicts 78% of malaria incidence. In addition, Model D predicts one month earlier than the start of the high malaria transmission season (rainy season and at the beginning of the dry season) and uses both the TCI11-13, which corresponds to the period of March 15th to April 15th, and the VHI49-52, which corresponds to the month of December (Fig. 7). The prediction of this model is given in the second half of March to April 15th and in the second half of December (Fig. 7). Considering Model A, which forecasts 75% of the incidence of malaria, malaria prediction is given in May corresponding to mosquitoes’ start activities (beginning of the rainy season). Nizamuddin et al*.* [20] came to the same conclusion in their study carried out in the province of Tripura, India. Both models can be used in the study area, northern Benin. The two models complement each other when we consider the variation in the season. Vegetation health is predicted in both the warm and wet seasons.

The most accurate model for predicting malaria incidence in northern Benin is the support vector machine model (Model D). Due to its emphasis on optimizing the margin between various classes and its contribution to avoiding overfitting the model, this model is less susceptible to outliers. SVM has the capacity to anticipate the onset of malaria activities (during the wet season and at the start of the dry season) by one month. In contrast to SVM, the Ordinary Least Squares (Model A) model offers coefficients for each predictor variable, making it simple to assess their impact on the outcome variable. Otherwise, Model A forecasts well the incidence or the number of cases of malaria in the northern part of Benin using only the weekly vegetation condition index (VCI) for weeks 19 to 21 in May. Model A can help to take earlier actions before the beginning of mosquito activities in May. Model B is best at providing preventive information in the dry season. Understanding malaria transmission in northern Benin requires careful monitoring of the state of vegetation. The ability of machine learning models such as SVM and OLS to forecast the occurrence of malaria and guide preemptive measures is demonstrated.

## Conclusion

In light of these findings, the availability of real-time vegetation health (VH) indices obtained from satellite images can be a useful tool in anticipating malaria epidemics and putting appropriate preventive measures into place. The VH indices, such as those offered by the AVHRR sensor on NOAA polar orbiting satellites, can assist in tracking changes in environmental factors that influence mosquito reproduction and survival, the main vectors of malaria. By having access to these data, public health professionals and other interested parties may better prepare for and respond to possible malaria outbreaks, especially in high-risk regions such as Northern Benin. The availability of such information via online resources such as the NOAA/NESDIS website can significantly help prevent and manage malaria, which will ultimately lead to better health outcomes.

## Data Availability

Data are available at the West African Science Service Centre on Climate Change and Adapted Land Use (WASCAL), Université de Lomé. You should contact the corresponding author for the data request.

## References

[1] Adefemi K, Awolaran O, Wuraola O (2015). Social and environmental determinants of malaria in under five children in Nigeria: a review. Int J Community Med Public Health.

[2] Cibulskis RE, Aregawi M, Williams R, Otten M, Dye C. Worldwide incidence of malaria in 2009: estimates, time trends, and a critique of methods. PLoS Med. 2011;e1001142 10.1371/journal.pmed.1001142 PMC3243721 22205883

[3] Mathanga DP, Tembo AK, Mzilahowa T, Bauleni A, Mtimaukenena K, Taylor TE (2016). Patterns and determinants of malaria risk in urban and peri-urban areas of Blantyre. Malawi Malar J.

[4] Endo N, Eltahir EAB (2016). Environmental determinants of malaria transmission in African villages”. Malar J.

[5] Leal Filho W, May J, May M, Nagy GJ (2023). Climate change and malaria: some recent trends of malaria incidence rates and average annual temperature in selected sub-Saharan African countries from 2000 to 2018. Malar J.

[6] WHO (2022). World malaria report 2022.

[7] Machault V. Utilisation de données d’observation de la terre par satellite pour l’évaluation des densités vectorielles et de la transmission du paludisme. Thesis. Faculté de Médecine de Marseille. 2010. https://www.redgems.org/sites/redgems.org/IMG/pdf/THESE_VANESSA_MACHAULT.pdf. Accessed 13 June 2023

[8] Enquête Démographique et de Santé. Bénin, 2017–2018. https://www.ilewaa.org/wp-content/uploads/2023/03/1.Benin-EDSBV-Rapport-final.pdf. Accessed 25 Dec 2022

[9] Odhiambo JN, Kalinda C, MacHaria PM, Snow RW, Sartorius B (2020). Spatial and spatio-temporal methods for mapping malaria risk: a systematic review. BMJ Glob Health.

[10] Thomson MC, Connor SJ (2001). The development of malaria early warning systems for Africa. Trends Parasitol.

[11] Kim HC, Pang S, Je HM, Kim D, Bang SY (2003). Constructing support vector machine ensemble. Pattern Recognit.

[12] World Bank. Climate change knowledge portal for development practitioners and policy makers. 2021. https://climateknowledgeportal.worldbank.org. Accessed 12 Dec 2022

[13] WHO (2021). World malaria report.

[14] Kogan F. Remote sensing for malaria: monitoring and predicting malaria from operational satellites. Springer Remote Sensing. 2020.

[15] Kogan F. 1981–2019 Vegetation Health Trends Assessing Malaria Conditions During Intensive Global Warming. In: Kogan F (ed.). Remote sensing for malaria: monitoring and predicting malaria from operational satellites. Springer Remote Sensing. 2020.

[16] Kasasa S, Asoala V, Gosoniu L, Anto F, Adjuik M, Tindana C (2013). Spatio-temporal malaria transmission patterns in Navrongo demographic surveillance site, northern Ghana. Malar J.

[17] Rahman A, Krakauer N, Roytman L, Goldberg M, Kogan F (2010). Application of Advanced Very High Resolution Radiometer (AVHRR)-based vegetation health indices for estimation of malaria cases. Am J Trop Med Hyg.

[18] Nizamuddin M, Kogan F, Dhiman R, Guo W, Roytman L (2013). Modeling and forecasting malaria in Tripura, India using NOAA/AVHRR-based vegetation health indices. Int J Remote Sens Appl.

[19] Thenkabail PS, Gamage MS, Smakhtin VU. The use of remote sensing data for drought assessment and monitoring in Southwest Asia. International Water Management Institute, Research Report 85. 2004.

[20] Kogan F, Popova F, Singh R, Alexandrova P (2018). Early forecasting corn yield using ground truth data and vegetation health indices in Bulgaria. Bulgar J Agricult Sci.

[21] Jensen R. Remote sensing of the environment: an earth resource perspective. Upper Saddle River, NJ : Prentice Hall, 2000.

[22] Lowe R, Chirombo J, Tompkins AM (2013). Relative importance of climatic, geographic and socio-economic determinants of malaria in Malawi. Malar J.

[23] Ayanlade A, OlugbadeAdeoye N, Babatimehin O (2013). Intra-annual climate variability and malaria transmission in Nigeria. Bull Geogr Socioeconom Ser.

[24] Gupta AK, Singh V, Mathur P, Travieso-Gonzalez CM. Prediction of COVID-19 pandemic measuring criteria using support vector machine, prophet and linear regression models in Indian scenario. Journal of Interdisciplinary Mathematics, 2021;24(1):89–108. 10.1080/09720502.2020.1833458

